# 

**Published:** 1866-07

**Authors:** 


### CONTENTS.

### ORIGINAL CONTRIBUTIONS.

ART. XXII. Practical Remarks on Blood-Letting. By D. B. Trimble, M.D., Chicago, 385

ART. XXIII. On the Use of Belladonna in Cholera. By J. A. Young, M.D., Monmouth, Ill., 397

ART. XXIV. Case of Vesuo-Vaginal Fistula—Operation on. By Darius Mason, M.D., Prairie du Chien, Wis., 400

### PROCEEDINGS OF SOCIETIES.

Proceedings of the Several Sections of the American Medical Association, during the recent Meeting in Baltimore, 402
Discussion on Diphtheria, 402
Inhalation of Nebulized Fluids, 406
Section on Medical Jurisprudence, Physiology, and Hygiene, 409
Report on Disenfectants, 410
Influence of Electricity on Epidemics, 410
The Use of Permanganate of Potash, 411
Compulsory Va cination, 411
On Alcohol and its Relations to Man, 413
Section on Surgery, 413
Four Operations for Lithotomy on Same Patient, 415
Section of Meteorology, Medical Topography, and Epidemic Diseases, 416
By-Laws of the Section of Meteorology, Medical Topography, and Epidemics, 417
Section on Psychology, 420
Proceedings of the Morgan County Medical Society, 420
Sixteenth Anniversary Meeting of the Illinois State Medical Society, 423

### THE CLINIQUE.

Clinical Cases in the Medical Wards of Mercy Hospital, June 5, 1866. By N. S. Davis, M.D., Prof. Practical and Clinical Medicine, Chicago Medical College, 433

### SELECTIONS.

On Ether as a Local Application. By John J. Black, M.D., Philadelphia, 438
On Dilitation of the Os Uteri during Labor by Incisions, 442
Treatment of Diphtheria, 443
Syphilitic Sarcocele. Sloughing of the Scrotum. Removal of the Gland. Nitrous-Oxide, 444
The Hydrate of Lime, 444

### EDITORIAL.

Mortality of the City of Chicago for April, 1866, 445
Personal, 446
Explanation, 446

